# Supplementary material for: Crossover in aromatic amino acid interaction strength between tyrosine and phenylalanine in biomolecular condensates
Source: eLife. 2025 Dec 4;14:RP104950. doi: 10.7554/eLife.104950 (PMC12677899; doi:10.7554/eLife.104950)
Supplement: Supplementary file 1. [file elife-104950-supp1.pdf]

Crossover in Aromatic Amino Acid Interaction Strength: Tyrosine vs. Phenylalanine in Biomolecular Condensates

David De Sancho[1,*] and Xabier López[1,†]

[1]*Polimero eta Material Aurreratuak: Fisika, Kimika eta Teknologia, Kimika Fakultatea, UPV/EHU & Donostia International Physics Center (DIPC), PK 1072, 20018 Donostia-San Sebastian, Euskadi, Spain*

Supporting Tables

System	Number of G/S/F-Y residues
GSY	300 / 300 / 300
GSF	300 / 300 / 300
GSY REMD	145 / 145 / 145
GSF REMD	145 / 145 / 145

TABLE I: Copies of each of the terminally capped amino acid residues in the condensate simulations. For GSY and GSF condensates, we use an equimolar mixture of all three components.

System	Number of molecules
Cyclohexane	500
Benzene	500
Toluene	500
Methanol	1000
Ethanol	1000
Hexanol	500
Octanol	500
Acetone	1000

TABLE II: Number of molecules used in pure solvent simulations for the determination of dielectric constants.

System	Simulation Time (μs)
GSY slab	3×1
GSF slab	3×1
GSY slab TIP4P-Ew	1
GSY slab REMD	48×1
GSF slab REMD	48×1
Solvents	0.5

TABLE III: Simulation times for each of the families of systems considered in this study. For GSY and GSF condensates, we run triplicates. For the simulations in pure solvents (including water), a single simulation run was performed for each system.

*Electronic address: `david.desancho@ehu.eus`
†Electronic address: `xabier.lopez@ehu.eus`
